# Polysaccharide Supplements from *Millettia speciosa Champ. ex Benth* Enhance Growth and Meat Quality in Wenchang Chickens

**DOI:** 10.3390/biology14070755

**Published:** 2025-06-24

**Authors:** Yu-Hang Liu, Jie Liu, Xin Feng, Quan-Wei Liu, Rui-Ping Sun, Wei Wu, Kun Ouyang, Jing-Li Yuan, Yan Zhang, Xiu-Ping Wang, Gui-Ping Zhao, Li-Min Wei

**Affiliations:** 1 Sanya Institute, Hainan Academy of Agricultural Sciences (Hainan Experimental Animal Research Center), Sanya 572025, China; lyh5621744@163.com (Y.-H.L.); jieliu2303@163.com (J.L.); xmxpww@163.com (W.W.); 15079542301@163.com (K.O.); 13250732023@163.com (J.-L.Y.); zhaoguiping@caas.cn (G.-P.Z.); 2 Hainan Key Laboratory of Tropical Animal Breeding and Epidemic Research, Institute of Animal Husbandry & Veterinary Research, Hainan Academy of Agricultural Sciences, Haikou 571100, China; lqw502@126.com (Q.-W.L.); ruiping937@126.com (R.-P.S.); zy79818_0@163.com (Y.Z.); 3 School of Life Science and Engineering, Foshan University, Foshan 528051, China; 3haofx@163.com; 4 Key Laboratory of WenChang Chicken Breeding and Feeding, Hainan (Tan Niu) Wenchang Chicken Co., Ltd., Haikou 571100, China; 562856433@163.com

**Keywords:** *Millettia speciosa Champ* polysaccharide, growth performance, meat quality

## Abstract

The polysaccharide of *Millettia speciosa Champ. ex Benth* (MSCP) has antioxidant properties, but its impact on chicken growth and development is not yet known. This study used chlortetracycline as a control to assess MCSP as a feed antibiotic substitute and its effects on Wenchang chicken production, slaughter performance, and meat quality. A total of 576 healthy 80-day-old Wenchang chickens were randomly allocated to six experimental groups. The control group (Control) received a basal diet, while the antibiotic group (CTC) was supplemented with 2 g/kg chlortetracycline (CTC). Four additional experimental groups were administered varying doses of MSCP: 400 mg/kg (MSCP_400_), 800 mg/kg (MSCP_800_), 1600 mg/kg (MSCP_1600_), and 3200 mg/kg (MSCP_3200_), respectively. The study discovered that incorporating MSCP and CTC into chicken diets significantly boosted the final body weight and average daily feed intake compared to the control group (*p* < 0.01), with MSCP notably enhancing average daily weight gain. With the addition of 800 mg/kg MSCP, chicken growth performance is comparable to that achieved with antibiotics in feed. However, it did not impact slaughtering performance (*p* > 0.05). In addition, MSCP significantly raised the pH after 24 h (*p* < 0.05) and decreased the yellowness (*p* < 0.01) of breast muscle. MSCP increased the essential amino acids (EAA) proportion in breast muscle (*p* < 0.05), EAA to nonessential amino acids (NEAA) ratio (*p* < 0.05), and diversity of fatty acids while decreasing C20:0 and increasing C18:2N6 and C22:1N9 content. Moreover, MSCP significantly reduced muscle fiber size (*p* < 0.01), increased fiber density (*p* < 0.01), boosted MYOD1 expression (*p* < 0.05), and decreased MSTN expression in breast muscle (*p* < 0.01). Overall, our study showed that supplementing the diet with MSCP, particularly at a dose of 800 mg/kg, enhanced growth, meat quality, muscle morphology, amino acid content, fatty acid composition, and gene expression related to muscle development in breast muscle. The results indicate that MSCP is a feed additive with the potential to replace antibiotics and improve meat quality, showing promising application potential.

## Simple Summary

The polysaccharide of *Millettia speciosa Champ. ex Benth* (MSCP) has antioxidant properties, but its impact on chicken growth and development is not yet known. This study used chlortetracycline as a control to assess MCSP as a feed antibiotic substitute and its effects on Wenchang chicken production, slaughter performance, and meat quality. A total of 576 healthy 80-day-old Wenchang chickens were randomly allocated to six experimental groups: a control group (Control), an antibiotic group (CTC), and four additional experimental groups administered varying doses of MSCP: 400 mg/kg, 800 mg/kg, 1600 mg/kg, and 3200 mg/kg, respectively. The study that incorporated MSCP and CTC into chicken diets significantly boosted the final body weight and average daily feed intake compared to the control group, with MSCP notably enhancing average daily weight gain. With the addition of 800 mg/kg MSCP, chicken growth performance is comparable to that achieved with antibiotics in feed. In addition, adding MSCP, especially in 800 mg/kg, to the diet improved meat quality, muscle morphology, and muscle development gene expression, certain amino acid content, and fatty acid composition in breast muscle. The results indicate that MSCP is a feed additive with the potential to replace antibiotics and improve meat quality, showing promising application potential.

## 1. Introduction

As living standards improve, people’s dietary requirements are also increasing, particularly when it comes to meat products, where they place greater emphasis on nutritional value and health benefits. Chicken, a high-protein, low-cholesterol meat, is favored for its rich amino acid content and unsaturated fatty acids [1,2,3]. Research indicates that the protein in chicken is not only easy to digest but also has an amino acid profile that closely matches the ideal ratio required by the human body, making chicken an excellent source of high-quality protein [4].

Moreover, chicken contains a variety of B vitamins, including thiamine, vitamin B6, and pantothenic acid, which play crucial roles in energy metabolism and nervous system function [2,5]. Chicken is also rich in minerals such as iron, zinc, and copper, which are essential for maintaining human health [6]. Epidemiological studies worldwide have shown a significant link between chicken consumption and good health, particularly in preventing overweight, obesity, cardiovascular disease, and type 2 diabetes [4].

Phytogenic feed additives (PFAs) have emerged as a promising alternative to antibiotics in animal production, offering multiple benefits such as enhanced growth performance, improved meat quality, and reduced reliance on synthetic growth promoters [7,8]. These natural compounds, derived from plants, herbs, and spices, are increasingly recognized for their potential to improve animal health and productivity, while addressing consumer concerns about antibiotic use in livestock.

*Millettia speciosa Champ.* ex *Benth* (*M. speciosa*) is a plant known for its medicinal and nutritional properties, particularly in traditional Chinese medicine [9]. The roots of *M. speciosa* are rich in flavonoids and polysaccharides, which contribute to its various biological activities. Research has shown that the flavonoid-enriched extract from *M. speciosa* can prevent obesity by regulating thermogenesis and lipid metabolism in high-fat diet-induced obese mice. This extract was found to reduce body weight gain, liver weight gain, and lipid accumulation, while also promoting thermogenesis in brown adipose tissue and activating lipolysis and fatty acid oxidation in white adipose tissues [10].

Additionally, the polysaccharides from *M. speciosa* (MSCP) have demonstrated protective effects against cyclophosphamide-induced intestinal injury and immunosuppression in mice. The polysaccharides were able to restore intestinal morphology, improve the number of goblet cells, and enhance the expression of genes related to intestinal mucosal integrity. They also improved microbial community diversity and regulated the relative abundance of dominant microbiota, suggesting their potential as a promising immunostimulant in functional foods and drugs [11]. Meanwhile, MSCP improved intestinal structure, increased villus height/crypt depth (V/C) ratio, and enhanced goblet cell and mucin expression. The immunomodulatory assay showed that MSCP can enhance pinocytic capacity and increase nitric oxide (NO) and cytokine secretion by regulating mRNA expression in RAW264.7 cells [12]. Although MSCP has not been studied in chickens, its known benefits in other species—such as antibacterial, anti-inflammatory, antioxidant, and intestinal development properties—indicate it could be a viable antibiotic alternative and promote broiler chicken growth.

Wenchang chicken, as an important local chicken breed, has attracted attention for its adaptability in tropical environments and excellent meat quality [13]. In research, Wenchang chicken is widely used as a model for anti-infection and growth promotion studies, mainly due to its unique performance in growth and immune function [14,15]. This study used chlortetracycline as a control to assess MCSP as a feed antibiotic substitute and its effects on Wenchang chicken production, slaughter performance, and meat quality.

## 2. Materials and Methods

### 2.1. Test Material

The experimental chicken came from Hainan (Tanniu) Wenchang Chicken Co., Ltd. (Haikou, China). Chlortetracycline hydrochloride, HY-B1327, came from MedChemExpress. MSCP, with 50% purity, was sourced from Xian QuanAo Biotech Co., Ltd. in Shanxi, China (Supplementary Table S1).

### 2.2. Experimental Design

A total of 576 Wenchang chickens at the age of 80 days were randomly assigned to 6 groups; each group contained 8 pens per treatment and 12 birds per pen. The control group (Control group) was fed with a basal diet, the antibiotic group (CTC) was supplemented with 2 g/kg CTC, and the experimental group was supplemented with 400 mg/kg MSCP (MSCP_400_ group), 800 mg/kg MSCP (MSCP_800_ group), 1600 mg/kg MSCP (MSCP_1600_ group), and 3200 mg/kg MSCP (MSCP_3200_ group). The composition and nutrient level of diets are shown in Table 1. The basal diet was designed to meet the nutrient requirements recommended for yellow chickens (NY/T3645-2020) [16]. The feeding experiment took place at Yongfa Base, part of the Hainan Academy of Agricultural Sciences. The experiment’s chickens were housed in a three-tier cage with feed and water, exposed to both artificial and natural light, and fed twice daily, morning and afternoon. The temperature and humidity during feeding had to follow the regulations of yellow-feathered broilers (NY/T1871-2010) [17]. Immunization followed the standard procedure with a 40-day experimental period. The chicken was euthanized by injecting pentobarbital sodium (150 mg/kg) into the vein, following the 2020 AVMA Guidelines, and then weighed. The Experimental Animal Ethics Committee of the Animal Husbandry and Veterinary Research Institute, Hainan Academy of Agricultural Sciences, approved all experimental procedures in this study on 3 February 2023 (HNSYY20230203).

### 2.3. Growth Performance Testing 

The feed consumption, initial body weight (IBW), and final body weight (FBW) of the chickens during the experiment were recorded in each repetition, and the average daily feed intake (ADFI), average daily gain (ADG) and feed conversion rate (FCR) were calculated. The calculation formula is as follows:

ADG = (FBW − IBW)/Trial days

ADFI = Total feed intake/Trial days

FCR = ADFI/ADG

### 2.4. Slaughtering Performance Testing

At the end of the experiment, one chicken from each pen was chosen at random, weighed, and euthanized by bleeding. The weights of the carcass, semi-clean body, fully eviscerated body, breast muscle, and leg muscle were measured using the guidelines in “Poultry Production Performance Terminology and Measurement Statistical Method” (NY/T823/2020) [18,19], along with dressing percentage, semi-eviscerated percentage, eviscerated percentage, breast muscle percentage, thigh muscle percentage, and abdominal fat rate. The dressed percentage of slaughter rate is the percentage of dressed weight to live weight before slaughter. The dressed weight is the weight of poultry after bloodletting, removal of feathers, cuticles, toe shells, and beak shells.

### 2.5. Meat Quality

To assess meat quality, we gathered the full chest and leg muscles from chickens bred for slaughter. The method for determining quality is as follows:

(1) The color of the breast and thigh muscles is measured within 45 min of slaughter using a spectrophotometer (TS7700, 3nh, Shenzhen Threenh Technology co., LTD, Shenzhen, China). The average color value is calculated after three repeated measurements.
(2) The pH of the breast and thigh muscles is measured 45 min and 24 h after slaughter using a Testo 205 pH meter, with the average value calculated from three repeated measurements.
(3) To calculate cooking loss, we trimmed the meat to 2.0 cm × 1 cm × 0.5 cm, recorded its initial weight (*m*1), wrapped it in foil, heated it in a water bath, let it cool and absorb the surface moisture, and recorded the final weight (*m*2). The calculation formula is as follows:

Cooking loss (%) = 100 × (*m*1 − *m*2)/*m*1.

(4) To measure drip loss, we trimmed the meat to 2.0 cm × 1 cm × 0.5 cm and recorded the weight as *m*1. The meat sample was suspended in a conical bottle with a thin thread. The meat sample could not touch the conical bottle. The mouth of the bottle was sealed by self-sealing film and placed in the refrigerator at 4 °C. After 24 h, the liquid on the surface of the meat sample was dried with filter paper and weighed as *m*2, and the dripping loss was calculated. The calculation formula is as follows:

Drip loss (%) = 100 × (*m*1 − *m*2)/*m*1.

(5) A 4.0 cm × 1 cm × 0.5 cm meat column was stripped of tendons, fat, and sarcolemma, and cut vertically with a muscle tenderness meter (C-LM36, Tenovo, Beijing, China) to measure shear force. This process was repeated 10 times and the average value is recorded.

### 2.6. Muscle Hematoxylin and Eosin (H&E) Staining

Immediately after slaughter, the right breast muscle (near the keel) was collected and fixed in 4% formaldehyde solution. Hematoxylin and eosin staining was used to examine the muscle morphology. ImagePro Plus 6.0 analysis software was used to measure the number and total area of muscle fibers in each section and to calculate the diameter of muscle fiber (μm) and density (number of roots/mm^2^).

### 2.7. Routine Muscle Nutrient Testing

The levels of nutrients, protein, and fat in breast muscle are determined by GB 5009.5-2016 and GB 5009.6-2016 [20,21]. To determine moisture content, remove fascia, and adipose tissue from the sample, we cut 30 g of meat, weighed it as A1, dried it at 105 °C for 16 h, weighed it as A2, dried it again for 2 h, then weighed it as A3. If |A3 − A2| ≤ 0.01 g, the moisture was considered dried. The moisture content is calculated as follows:

Moisture = A1 − A3/A1

### 2.8. Detection of Amino Acid Contents in Muscle

Referring to the method of QI [22], approximately 30 mg of freeze-dried samples were hydrolyzed in 6 M of hydrochloric acid at 110 °C for 24 h. The suspension was diluted with water, and 1 mL of the sample was filtered using a 0.2 µm filter. The samples were analyzed using an HPLC-based automatic amino acid analyzer (Ultimate3000-API 3200 Q TRAP; ThermoFisher Scientific, Waltham, MA, USA), according to the manufacturer’s instructions. The contents of amino acids in the muscle were expressed as mg/g of dried tissue.

### 2.9. Detection of Fatty Acid Contents in Muscle

The left breast muscle was frozen at −20 °C, extracted, and analyzed for fatty acid content using a method outlined in (GB 5009.168/2016) [23] using ultra-high performance liquid chromatography (LC20, Shimadzu, Japan). The content and types of fatty acids are calculated according to the content of fatty acid methyl ester and conversion coefficient.

### 2.10. mRNA Expression Analysis

We extracted total RNA from the breast muscle using the RNA Easy Fast Total RNA Extraction Kit (TIANGEN, Beijing, China), determined the concentration and purity with Ultramicro Spectrophotometer (IMPLEN P330, Munich, Germany), reverse transcribed and synthesized cDNA following instructions from the Reverse Transcription Kit (TIANGEN, Beijing, China), and stored it at −20 °C. The real-time qPCR detection system underwent pre-denaturation at 95 °C for 5 min, followed by denaturation at 95 °C for 30 s, annealing at 60 °C for 30 s, and extension at 72 °C for 15 s for 40 cycles. β-actin was used as the internal reference gene to quantify the mRNA expression of *MYOG*, *MYOD1,* and *MSTN* in breast muscle using the 2-quantitative CT method with primer sequence, synthesized by Beijing Tsingke Biotechnology Co., Ltd. (Beijing, China), as shown in Table 2.

### 2.11. Statistical Analysis

We analyzed the data using SPSS version 26.0 (IBM Corp., Chicago, IL, USA). The data was expressed as “average ± standard deviation”. One-way ANOVA was used to analyze the differences between groups, *p* < 0.05 showed significant difference, while *p* < 0.01 showed extremely significant difference.

## 3. Results

### 3.1. Growth Performance

As shown in Table 3, the FBW and ADFI of CTC, MSCP_400_, MSCP_800_, MSCP_1600_, and MSCP_3200_ groups were significantly higher than those of the control group (*p* < 0.01). The ADG of the MSCP group was significantly higher than the control group (*p* < 0.01), but no significant difference was observed between the control and CTC groups (*p* > 0.05). There were no significant differences found in FCR among groups (*p* > 0.05).

### 3.2. Slaughtering Performance

Table 4 shows that there is no significant effect on dressing percentage, semi-eviscerated percentage, eviscerated percentage, breast muscle percentage, thigh muscle percentage, and abdominal fat rate among the groups (*p* > 0.05).

### 3.3. Meat Quality Traits

As shown in Table 5 and Table 6, the pH_24 h_ of breast muscle was significantly higher in the MSCP_400_ and MSCP_800_ groups compared to the control group (*p* < 0.05). The cooking loss of thigh muscle in the MSCP_400_ group was significantly lower than in the other groups (*p* < 0.01). The shearing force of thigh muscle in the MSCP_800_ and MSCP_1600_ groups decreased significantly compared to the control group (*p* < 0.05). The b* of breast muscle in MSCP groups was significantly lower than that in the control and CTCCTC groups (*p* < 0.01). No significant differences in breast and thigh muscle meat quality were found between the control and CTC groups, except for cooking loss in the thigh muscle (*p* > 0.05).

### 3.4. Conventional Intramuscular Nutrients

As shown in Table 7, the moisture of breast muscle increased significantly in the MSCP_400_, MSCP_1600_, and MSCP_3200_ groups compared to the CTC (*p* < 0.05). The content of crude protein in breast muscle of the CTC and MSCP_400_ groups was significantly lower than in other experimental groups (*p* < 0.05). The CTC showed significantly higher intramuscular fat content in breast muscle than the other groups (*p* < 0.01).

### 3.5. Amino Acid Contents in Muscle

Table 8 demonstrated that the citrulline content in the MSCP_800_ group was significantly higher than in other groups (*p* < 0.05) (Supplementary Table S2). The proportion of EAA in the MSCP_800_ group was significantly higher than that in Control group, MSCP_400_, MSCP_1600_ and MSCP_3200_ group (*p* < 0.05). The EAA/TAA and EAA/NEAA ratios were also higher in the MSCP_800_ group compared to the control, MSCP_400_, MSCP_1600_, and MSCP_3200_ groups (*p* < 0.05). There were no notable differences in TAA, EAA, NEAA, FAA, EAA/TAA, and EAA/NEAA of breast muscle between the control and CTCs (*p* > 0.05).

### 3.6. Fatty Acid Contents in Muscle

Table 9 showed 25 fatty acid (FA) species, including 12 saturated fatty acids (SFA) and 13 unsaturated fatty acids (UFA). The CTC had significantly higher levels of lauric acid compared to the control group (*p* < 0.05) for SFAs. The content of arachidonic acid (C20:0) in MSCP_1600_ group was significantly lower than that in the MSCP_400_, MSCP_800_, MSCP_3200_ and CTC (*p* < 0.01) (Supplementary Table S3). The content of lignin acid (C24:0) in the control group was significantly lower than in other experimental groups (*p* < 0.01) (Supplementary Table S3). The content of heptadecanoic acid (C17:0) in the MSCP_1600_ group was significantly lower than in the control and MSCP_800_ group (*p* < 0.05) (Supplementary Table S3). The content of tridecanoic acid (C13:0) in the MSCP_800_, MSCP_1600_ and MSCP_3200_ group was significantly lower than in the CTC and control group (*p* < 0.05) (Supplementary Table S3). For polyunsaturated fatty acids (PUFAs), the content of linoleic acid (C18:2n6) in the MSCP_800_ group was significantly higher than in the control and MSCP_1600_ group (*p* < 0.05). Erucic acid (C22:1n9) was found in MSCP_800_ and MSCP_1600_ groups, with no significant difference in content between the two groups (*p* > 0.05). No significant impacts were observed on PUFAs, SFAs, MUFAs, PUFAs/SFAs or n-6/n-3PUFAs ratios among groups (*p* > 0.05).

### 3.7. Muscle Morphology

**Figure 1:**
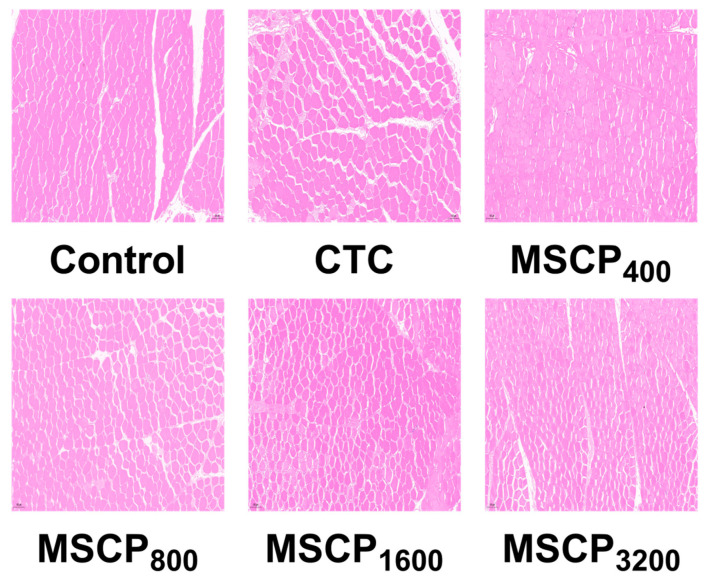
Muscle tissue morphology. Scale bars: 100 μm.

As shown in Table 10, the experimental group had a significantly smaller muscle fiber diameter than the control group (*p* < 0.01). Additionally, the density of pectoralis muscle fiber in the MSCP test group significantly increased (*p* < 0.01), as illustrated in Figure 1. The CTC showed a significantly higher muscle fiber diameter and lower density root compared to the control group (*p* < 0.01).

### 3.8. Muscle Development-Related Genes Expression

**Figure 2:**
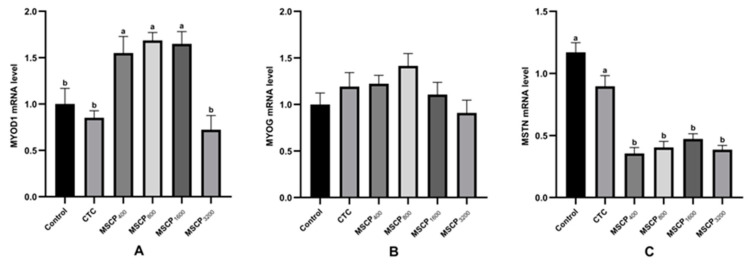
Muscle differentiation-related genes expression. (**A**–**C**) The *MYOD1* (**A**), *MYOG* (**B**), and *MSTN* (**C**) mRNA expressions of breast muscle. Different superscript letters indicate significant differences (*p* < 0.05). Data are presented as the mean ± SD. The number of samples in each group is 8.

In breast muscle, *MYOD1* mRNA expression was significantly higher in the MSCP_400_, MSCP_800_, and MSCP_1600_ groups than in the control group (*p* < 0.05). There are no significant differences in *MYOG* mRNA expression among the groups. *MSTN* mRNA expression was significantly lower in the MSCP test group compared to the control and CTCs (*p* < 0.01) (Figure 2).

## 4. Discussion

This study explored using MSCP as an antibiotic substitute in broiler feed and its effects on chicken growth, meat quality, and muscle development. CTC is a tetracycline with broad-spectrum antibacterial activity, used for the treatment of rickettsia, mycoplasma, spirochete, and other bacterial infections [24]. CTC is commonly used in animal agriculture for disease prevention and treatment, particularly in improving broiler production performance as a premix [25]. MSCP exerts its biological activity through various mechanisms, such as immune regulation, enhanced intestinal barrier function, and regulation of gut microbiota [11,26]. We suggest that MSCP may function somewhat like CTC.

Plant polysaccharides are an important plant active ingredient [27]. Jiang et al. [28] discovered that adding *Gracilaria lemaneiformis* polysaccharides to broiler diets increased growth rate and improved intestinal health, leading to better overall performance. Zhu et al. found that adding *Pseudostellaria heterophylla* polysaccharide to Gushi chickens’ diets can improve their growth performance and reduce FCR [29]. The study showed that MSCP increased chicken growth without impacting feed efficiency. The best improvement was seen with 800 mg/kg of MSCP. MSCP had similar growth-promoting effects as antibiotics, with no significant difference in growth between MSCP and CTCs.

Dressing percentage is an important index to measure slaughtering performance. It is generally believed that a slaughtering percentage of chickens above 80% indicates good meat quality [30]. This study found that each group of Wenchang chickens had a dressing percentage of over 90%, indicating good meat performance. However, neither MSCP nor CTC affects the slaughter performance of Wenchang chickens. pH, texture, and muscle color are key factors in assessing the quality of chicken meat [31]. Higher a* values in meat indicate better quality, as shown by lower L* and b* values. Shear force is a key factor in assessing meat tenderness, with low shear force and good quality meat indicating good water retention capacity in muscle [32]. Muscle pH affects water binding capacity. Higher levels of lactic acid in muscles decrease pH, reducing the ability of muscles to bind water and protein, leading to increased drip loss. Maintaining a high pH can help muscles retain water [33,34,35]. In this study, we found that MSCP can reduce the b* of breast muscle. Moreover, MSCP increased breast muscle pH_24 h_ and reduced acidification, with the best improvement seen with the addition of 800 mg/kg to the diet. It is worth noting that the CTC has a relatively small impact on meat quality, far less than the 800 mg/kg MSCP group. One possible mechanism is that the antioxidant activity of MSCP protects the cell membrane of muscle cells and reduces their peroxidation damage. MSCP also reduces cooking loss and shear force in thigh muscle. We also analyzed the protein, moisture, and fat content in muscles to assess how MSCP affects meat quality [36]. It is generally believed that the muscle quality is best when moisture content is between 70 and 80% [37]. Protein levels impact nutritional value, while intramuscular fat affects flavor. A moderate amount of fat enhances meat flavor [38]. In this experiment, the water content of breast muscle in various groups is approximately 70%. Adding CTC in feed altered crude protein and crude fat levels in muscles, while MSCP had little effect. The above results showed that the addition of MSCP to the diet can improve meat quality and improve storage and processing outcomes.

Research has found that adding natural plant ingredients can enhance the amino acid content and flavor of meat [39,40]. Amino acid content is crucial for evaluating meat quality. Essential amino acids (EAAs) cannot be produced by the human body and must be consumed through food. Chicken is a good source of EAAs, which are essential for human metabolism [41]. In this experiment, adding MSCP to the diet increased citrulline content in breast muscle, promoting muscle protein synthesis and improving muscle function. A total of 800 mg/kg of MSCP can improve meat quality by increasing EAA/TAA and EAA/NEAA ratios, surpassing the amino acid ratio recommended by the World Health Organization for excellent meat. Similarly, the CTC had a relatively small impact on the composition of amino acids in the breast muscles.

The FA content in meat determines the nutritional value of meat, and meat with good FA composition is more likely to be accepted by people [42]. Lower levels of SFA and higher levels of UFA are more beneficial to human health [43]. A total of 25 kinds of FA are detected in breast muscle of Wenchang chicken, including 12 kinds of SFA and 13 kinds of UFA. Many studies have found that plant polysaccharides can change the content and composition of fatty acids in poultry.

For example, Lixiang et al. found that polysaccharides from Yingshan Yunwu tea (GTPS) can increase the total amount of free amino acids of breast meat, and increase the content of histidine, leucine, serine, glutamic acid, and alanine. GTPS also increased contents of inosine monophosphate and guanylic monophosphate, which improved the meat flavor of chickens [44].

This study found that MSCP raised linoleic acid levels and lowered heptadecanoic and tridecanoic acid levels in breast muscle, indicating it may be beneficial for human health. Moreover, erucic acid is only detected in the experimental group of MSCP, which indicated that MSCP not only improved the composition of fatty acids but also increased the diversity of unsaturated fatty acids. The CTC influences fatty acid content in chest muscle, but less effectively than the 800 mg/kg MSCP group. Our research results indicate that adding MSCP, especially at 800 mg/kg, to diets may increase unsaturated fatty acids in breast muscle, improving meat’s nutritional value.

Muscle tissue morphology reflects muscle development. Muscle histological characteristics include muscle fiber diameter, cross-sectional area, density, and other factors [45]. Studies have shown that muscle tenderness is influenced by the diameter and density of muscle fibers. Smaller diameter and higher density result in more tender muscles [46,47]. The thoracic muscle fiber diameter was significantly lower in the MSCP group compared to the Control and CTCs, with the Control group also showing a significantly lower diameter compared to the CTC. In this way, we further studied the related genes that regulate muscle development. MYOD1 and MYOG are myogenic regulatory factors, and their expression level is positively correlated with muscle growth rate [48]. MYOD1 can not only promote muscle development, but also promote muscle typing, such as promoting the transformation of type I muscle fibers into type II muscle fibers [49,50]. MSTN is a key growth factor in animals found mainly in muscle tissue, where it negatively regulates muscle growth and development by inhibiting muscle progenitor cell proliferation [51,52]. In this study, MSCP significantly enhanced the mRNA expression of MYOD1 and decreased the expression of MSTN in breast muscle. MSCP may enhance muscle growth by influencing the expression of muscle-related genes, potentially through effects on nutrient absorption and digestive enzymes. Further research is needed to understand the mechanisms behind this regulation. Overall, adding MSCP to broiler diets can improve muscle tissue morphology and gene expression related to muscle development.

## 5. Conclusions

Our study found that adding MSCP, especially at a high dose of 800 mg/kg, to the diet improved FBW, meat quality, muscle morphology, and muscle development gene expression, certain amino acid content, and fatty acid composition in breast muscle. MSCP had growth-promoting effects similar to antibiotics and was more effective than CTC in improving meat quality in Wenchang chicken. The results indicate that MSCP is a feed additive with the potential to replace antibiotics and improve meat quality, showing promising application potential.

## Figures and Tables

**Table 1:** Composition and nutrient level of diets (dry matter basis, %).

Ingredients Composition (%)	Control
Corn	72.1
Bran	4.5
Soybean meal	17.1
Soybean oil	3.0
CaHPO_4_	1.0
Limestone	1.0
Premix *	1.0
NaCl	0.3
Nutrient level (dry mater basis, %)	
ME, MJ/kg	14.80
Crude protein	14.50
Crude Fat	6.04
Crude fiber	2.44
Calcium	0.69
Phosphorous	0.51
Available + Phosphorus	0.38
Lysine	0.65
Methionine	0.39
Arginine	0.87

* The premix provided the following per kg of diets per kg diet: Cu 10 mg, Fe 80 mg, Mn 60 mg, Zn 70 mg, I 2 mg, Se 0.40 mg, Vitamin A 10,000 IU, Vitamin E 10 mg, Vitamin B2 12 mg, Vitamin B6 23 mg, Vitamin B12 63.50 µg, nicotinic acid 15 mg, folic acid 0.50 mg, pantothenic acid 10 mg, biotin 0.15 mg, cyanocobalamin 10 μg. Metabolizable energy (ME) is calculated and the rest is measured.

**Table 2:** Real-time quantitative PCR primers.

Gene	Primer Sequences (5′→3′)	Accession	TM °C
*β-actin*	F: CATTGTCCACCGCAAATGCT R: AAGCCATGCCAATCTCGTCT	L08165.1	60
*MYOG*	F: TTTTCCCGGAGCAGAGGTTT R: GGTCGATGGACACGGTTTTG	NM_204184.2	60
*MYOD1*	F: GCCCTCGCTCCAACTGCTCC; R: GCTGCCTTTTGGAGTTTGCG	NM_204214.3	60
*MSTN*	F: TTTTGGATGGGACTGGATTATAGCACCT R: GCCTCTGGGATTTGCTTGGTGTACC	NM_001001461.2	60

**Table 3:** Effect of dietary MSCP supplementation on growth performance of Wenchang chickens.

Tratis ^1^	Groups ^2^						*p*-Value
	Control	CTC	MSCP_400_	MSCP_800_	MSCP_1600_	MSCP_3200_	
IBW/g	1476.04 ± 8.26	1476.04 ± 10.39	1473.96 ± 6.95	1475.00 ± 8.91	1475.00 ± 4.45	1478.12 ± 4.31	0.919
FBW/g	2055.42 ± 57.84 ^b^	2161.67 ± 29.91 ^a^	2127.29 ± 44.85 ^a^	2130.95 ± 39.30 ^a^	2120.00 ± 20.04 ^a^	2109.58 ± 74.22 ^a^	0.004
ADG/g	14.49 ± 1.56 ^b^	17.14 ± 0.79 ^ab^	16.33 ± 1.14 ^a^	16.87 ± 1.70 ^a^	16.41 ± 0.92 ^a^	15.79 ± 1.85 ^a^	0.007
ADFI/g	70.97 ± 9.42 ^b^	95.15 ± 0.75 ^a^	93.04 ± 3.13 ^a^	93.08 ± 0.86 ^a^	95.15 ± 10.50 ^a^	91.09 ± 1.83 ^a^	<0.001
FCR	5.59 ± 1.16	5.56 ± 0.27	5.55 ± 0.52	5.33 ± 0.30	5.58 ± 0.11	5.67 ± 0.39	0.942

^1^ Growth performance. IBW, initial body weight; FBW, final body weight; ADG, average daily gain; ADFI, average daily feed intake; FCR, feed conversion ratio. ^2^ CTC, chlortetracycline hydrochloride; MSCP, *Millettia speciosa Champ.* ex *Benth* polysaccharides. Different superscript letters indicate significant difference (*p* < 0.05). Data are presented as the mean ± SD. The number of samples in each group is 8.

**Table 4:** Effect of dietary supplementation of MSCP on slaughtering performance of Wenchang chickens.

Tratis ^1^	Groups						*p*-Value
	Control	CTC	MSCP_400_	MSCP_800_	MSCP_1600_	MSCP_3200_	
Dressing percentage%	92.73 ± 2.51	92.34 ± 1.67	93.39 ± 4.48	92.74 ± 4.62	94.57 ± 1.30	95.37 ± 1.46	0.297
Semi-eviscerated percentage%	79.95 ± 3.51	81.97 ± 4.63	83.46 ± 5.58	84.27 ± 3.02	81.34 ± 1.30	82.36 ± 1.40	0.298
Eviscerated percentage%	68.75 ± 3.84	69.46 ± 0.47	70.27 ± 3.63	71.39 ± 2.51	70.14 ± 2.16	70.72 ± 3.41	0.623
Breast muscle percentage%	12.35 ± 1.44	13.15 ± 0.96	13.01 ± 1.25	13.09 ± 1.34	13.67 ± 1.27	12.89 ± 1.67	0.585
Thigh muscle percentage%	16.53 ± 1.75	16.58 ± 1.44	16.35 ± 1.41	16.31 ± 1.93	16.89 ± 1.39	16.24 ± 2.71	0.986
Abdominal fat rate%	9.19 ± 1.88	9.32 ± 1.99	9.16 ± 1.85	8.51 ± 1.66	9.12 ± 1.63	7.59 ± 1.82	0.406

^1^ Slaughter performance. Data are presented as the mean ± SD. The number of samples in each group is 8.

**Table 5:** Effect of dietary MSCP supplementation on meat quality traits of breast muscle.

Tratis ^1^	Groups						*p*-Value
	Control	CTC	MSCP_400_	MSCP_800_	MSCP_1600_	MSCP_3200_	
pH_45 min_	5.97 ± 0.18	5.94 ± 0.23	6.20 ± 0.28	6.09 ± 0.30	6.00 ± 0.23	5.98 ± 0.10	0.389
pH_24 h_	5.82 ± 0.05 ^bc^	5.76 ± 0.15 ^c^	6.03 ± 0.06 ^a^	6.06 ± 0.20 ^a^	5.96 ± 0.19 ^ab^	5.96 ± 0.14 ^ab^	0.014
Drop loss at 24 h/%	2.10 ± 0.84	3.19 ± 0.49	2.31 ± 0.82	2.03 ± 0.42	2.08 ± 0.47	2.20 ± 0.28	0.090
Cooking loss/%	17.73 ± 5.21	16.40 ± 2.94	12.12 ± 1.26	15.05 ± 1.05	16.82 ± 1.50	17.52 ± 3.50	0.063
Shear force/N	14.16 ± 2.40	12.48 ± 2.23	13.02 ± 1.96	11.87 ± 2.14	11.67 ± 2.49	12.57 ± 2.04	0.452
Lightness (L *)	49.91 ± 1.86	48.66 ± 2.50	48.98 ± 3.05	47.96 ± 2.69	48.71 ± 2.33	48.99 ± 3.52	0.334
Redness (a *)	0.61 ± 0.15	0.83 ± 0.19	0.70 ± 0.22	0.81 ± 0.22	0.74 ± 0.34	0.67 ± 0.27	0.989
Yellowness (b *)	12.46 ± 2.37 ^a^	12.02 ± 1.51 ^a^	9.89 ± 2.24 ^b^	10.22 ± 1.76 ^b^	9.46 ± 2.76 ^b^	8.89 ± 2.99 ^b^	<0.001

^1^ Meat quality. Note: pH_45 min_, pH after 45 min. pH_24 h_, pH after 24 h. Different superscript letters indicate significant difference (*p* < 0.05). Data are presented as the mean ± SD. The number of samples in each group is 8.

**Table 6:** Effect of dietary MSCP supplementation on meat quality traits of thigh muscle.

Tratis ^1^	Groups						*p*-Value
	Control	CTC	MSCP_400_	MSCP_800_	MSCP_1600_	MSCP_3200_	
pH_45 min_	5.76 ± 0.27	5.95 ± 0.53	6.13 ± 0.36	6.28 ± 0.49	5.84 ± 0.30	5.80 ± 0.12	0.312
pH_24 h_	5.61 ± 0.22 ^b^	5.62 ± 0.02 ^b^	5.69 ± 0.13 ^ab^	5.71 ± 0.07 ^ab^	5.86 ± 0.23 ^a^	5.92 ± 0.13 ^a^	0.021
Drop loss 24 h/%	1.88 ± 0.44	1.93 ± 0.90	1.86 ± 0.69	2.30 ± 0.59	1.68 ± 0.65	1.94 ± 0.30	0.731
Cooking loss/%	27.10 ± 2.01 ^b^	31.66 ± 1.41 ^a^	21.42 ± 4.20 ^c^	28.34 ± 3.24 ^ab^	30.49 ± 2.55 ^ab^	30.53 ± 3.68 ^ab^	0.001
Shear force/N	29.53 ± 1.22 ^a^	28.52 ± 3.91 ^a^	28.55 ± 2.56 ^a^	23.77 ± 1.93 ^b^	23.55 ± 4.31 ^b^	29.61 ± 4.76 ^a^	0.015
Lightness (L *)	47.83 ± 2.68	49.69 ± 2.74	49.79 ± 2.80	48.13 ± 1.98	48.49 ± 2.97	49.54 ± 3.48	0.068
Redness (a *)	6.67 ± 3.56	7.30 ± 3.26	7.20 ± 2.21	8.28 ± 2.32	7.80 ± 2.20	7.24 ± 1.66	0.407
Yellowness (b* )	15.04 ± 3.63	15.49 ± 2.79	14.02 ± 2.92	14.43 ± 3.75	13.45 ± 3.45	12.69 ± 5.60	0.136

^1^ Meat quality. Note: pH_45 min_, pH after 45 min. pH_24 h_, pH after 24 h. Different superscript letters indicate significant difference (*p* < 0.05). Data are presented as the mean ± SD. The number of samples in each group is 8.

**Table 7:** Effect of dietary MSCP supplementation on routine nutritional composition of muscle of breast muscle in Wenchang chickens.

Tratis	Groups						*p*-Value
	Control	CTC	MSCP_400_	MSCP_800_	MSCP_1600_	MSCP_3200_	
Moiture (%)	68.69 ± 1.15 ^ab^	67.97 ± 1.21 ^b^	69.70 ± 0.79 ^a^	69.10 ± 1.17 ^ab^	69.34 ± 0.55 ^a^	69.91 ± 1.21 ^a^	0.036
Crude Protein (g/kg)	732.18 ± 95.73 ^a^	611.73 ± 139.61 ^b^	693.44 ± 94.09 ^b^	778.02 ± 114.36 ^a^	770.99 ± 34.54 ^a^	783.84 ± 16.44 ^a^	0.024
Intramuscular fat (%)	3.98 ± 1.47 ^b^	10.14 ± 2.69 ^a^	6.94 ± 2.01 ^b^	5.88 ± 2.86 ^b^	5.76 ± 3.20 ^b^	5.62 ± 2.05 ^b^	0.005

Different superscript letters indicate significant difference (*p* < 0.05). Data are presented as the mean ± SD. The number of samples in each group is 6.

**Table 8:** Effect of dietary MSCP supplementation on free amino acid composition of breast muscle in Wenchang chickens, ug/g.

Tratis	Groups						*p*-Value
	Control	CTC	MSCP_400_	MSCP_800_	MSCP_1600_	MSCP_3200_	
TAA	309.05 ± 137.50	341.25 ± 126.79	290.71 ± 188.43	433.21 ± 173.73	264.11 ± 116.44	274.79 ± 96.07	0.365
EAA	96.49 ± 42.16	122.40 ± 54.83	97.22 ± 71.43	165.52 ± 71.64	89.26 ± 46.40	93.09 ± 42.02	0.182
NEAA	212.56 ± 95.65	218.85 ± 72.77	193.50 ± 117.25	267.69 ± 102.33	174.85 ± 71.27	181.71 ± 55.22	0.508
FAA	179.13 ± 81.28	192.64 ± 69.01	160.76 ± 96.28	218.23 ± 82.99	146.50 ± 59.77	152.18 ± 46.97	0.554
EAA/TAA	0.31 ± 0.01 ^b^	0.35 ± 0.04 ^ab^	0.31 ± 0.02 ^b^	0.38 ± 0.02 ^a^	0.33 ± 0.04 ^b^	0.33 ± 0.04 ^b^	0.016
EAA/NEAA	0.46 ± 0.02 ^b^	0.54 ± 0.08 ^ab^	0.46 ± 0.09 ^b^	0.61 ± 0.04 ^a^	0.49 ± 0.09 ^b^	0.50 ± 0.08 ^b^	0.013

EAA: essential amino acids; NEAA: nonessential amino acids; TAA: total amino acids; FAA: free amino acids. In the same column, values with different letter superscripts mean a significant difference (*p* < 0.05). Different superscript letters indicate significant difference (*p* < 0.05). Data are presented as the mean ± SD. The number of samples in each group is 6.

**Table 9:** Effect of dietary MSCP on the content of fatty acids in breast muscle of Wenchang chickens, ug/g.

Tratis	Groups						*p*-Value
	Control	CTC	MSCP_400_	MSCP_800_	MSCP_1600_	MSCP_3200_	
PUFAs	3068.65 ± 290.76	3571.86 ± 405.99	3584.58 ± 325.14	3333.60 ± 753.02	3164.20 ± 434.07	3513.31 ± 239.02	0.223
MUFAs	206.63 ± 38.53	247.19 ± 43.22	242.60 ± 70.61	210.28 ± 113.58	235.16 ± 58.11	250.98 ± 58.80	0.796
SFAs	2481.39 ± 234.09	2938.60 ± 277.92	2848.23 ± 252.45	2706.92 ± 689.47	2633.64 ± 424.53	2862.52 ± 283.36	0.370
PUFAs/SFAs	1.23 ± 0.03	1.21 ± 0.06	1.26 ± 0.02	1.24 ± 0.04	1.21 ± 0.04	1.23 ± 0.05	0.342
n-6/n-3 PUFAs	1.32 ± 0.03	1.30 ± 0.07	1.34 ± 0.03	1.31 ± 0.03	1.29 ± 0.03	1.32 ± 0.05	0.434

SFAs: saturated fatty acids; MUFAs: monounsaturated fatty acids; PUFAs: polyunsaturated fatty acids. n-6/n-3, total omega 6 to total omega 3 fatty acid ratio. Data are presented as the mean ± SD. The number of samples in each group is 6.

**Table 10:** Effects of dietary MSCP supplementation on breast muscle histological traits of Wenchang chickens.

Tratis	Groups						*p*-Value
	Control	CTC	MSCP_400_	MSCP_800_	MSCP_1600_	MSCP_3200_	
Diameter/μm	69.71 ± 3.49 ^b^	73.82 ± 3.94 ^a^	66.39 ± 2.30 ^c^	65.56 ± 3.44 ^c^	66.07 ± 3.92 ^c^	65.88 ± 2.42 ^c^	<0.001
Density root/mm^2^	453.00 ± 49.18 ^b^	393.74 ± 42.12 ^c^	494.47 ± 43.69 ^a^	522.30 ± 63.85 ^a^	513.24 ± 65.49 ^a^	505.53 ± 55.44 ^a^	<0.001

Different superscript letters indicate significant difference (*p* < 0.05). Data are presented as the mean ± SD. The number of samples in each group is 8.

## Data Availability

The original contributions generated for this study are included in the article. Further inquiries can be directed at the corresponding author.

## Supplementary Materials

The following supporting information can be downloaded at: https://www.mdpi.com/article/10.3390/biology14070755/s1, Table S1: The sugars, phenolic acids, and flavonoids in MSCP. Table S2: MSCP supplementation on free amino acid composition of breast muscle in Wenchang chickens ug/g. Table S3: Effect of dietary MSCP on the content of fatty acids in breast muscle of Wenchang chickens ug/g.
