# Supplementary material for: Polysaccharide Supplements from Millettia speciosa Champ. ex Benth Enhance Growth and Meat Quality in Wenchang Chickens
Source: Biology (Basel). 2025 Jun 24;14(7):755. doi: 10.3390/biology14070755 (PMC12292069; doi:10.3390/biology14070755)
Supplement: Supplementary file 1 [file biology-14-00755-s001.zip › biology-3655680-supplementary.pdf]

biology

MDPI

Table S1. The sugars, phenolic acids, and flavonoids in MSCP.

Sugars (ug/mg)		Phenolic acids (ng/mg)		Flavonoids (ng/mg)	
Arabinose	0	Gallic acid	673.8	Catechin	196.3
Rhamnose	9.98	Phenylalanine	95.6	Genistein	11.7
D-Fructose	208.1	3,4-dihydroxybenzoic acid	7.6	Puerarin	0
D-galactose	0	Protocatechuic aldehyde	0	Baicalin	0
D-Glucose anhydrous	10.28	Catechin	0	Diosmin	0
d-sorbitol	0.89	Vanillic acid	0	Kaempferol	65.1
Inositol	0	Caffeic acid	0	Luteolin	283.1
Sucrose	24.86	Syringic acid	37.1	Rutinose	99.3
D - (+) - trehalose	0	Epicatechin	0	Daidzein	0
D - (+) - mannose	0.39	Vanillin	0	Naringin	2.1
Xylose	1.05	4-hydroxybenzoic acid	183.6	Icariin	0
L - (-) - fucose	0	p-Hydroxycinnamic acid	0	Chrysin	0
D-Glucuronic acid	188.66	Salicylic acid	49.8	Silybin	0
Galacturonic acid Hydrate	0	Ferulic acid	3.1	Isoquercitrin	0
D-glucose-6-phosphate	0	4-hydroxy-3,5-dimethoxycinnamic acid	0	Fisetin	0
Maltose	0	Benzoic acid	33.5	Isovitexin	0
6-phosphate trehalose	0	Trans-Cinnamic acid	0	Muxiangcao Glycoside	449.2
Stachyose	0	Hydrocinnamic Acid	0	Apigenin	0
Raffinose	0	Syringaldehyde	0	Astragaloside	0
Lactose	0			Biochanin a	0
Glucose	10.28			Daidzin	0
				Dihydroquercetin	7.5
				Epicatechin	22.7
				Formononetin	0
				Genistin	0
				Glycitein	0
				Glycitin	0
				Liquiritigenin	4.8
				Quercitrin	0
T				Vitexin	0
				Dihydromyricetin	0
				Naringenin	0
				Quercetin	0
				Kaempferol	0
				Dihydromyricetin	0

Table S2 MSCP supplementation on free amino acid composition of breast muscle in Wenchang chickens ug/g.

Items	Groups						*P*-value
	Control	CTC	$MSCP_{400}$	MSCP800	$MSCP_{1600}$	$MSCP_{3200}$	
EAA							
Met	10.26±3.47	12.77±6.13	13.88±10.72	19.92±9.35	9.34±3.82	10.73±4.53	0.135
Val	12.33±7.70	13.45±5.64	10.64±8.08	21.13±9.82	10.99±7.48	11.78±6.70	0.200
Lys	7.86±3.66	9.30±4.42	9.65±8.30	12.71±4.20	8.46±6.46	6.85±3.49	0.529
Ile	15.78±8.87	19.33±8.78	17.20±15.57	32.88±16.40	13.56±8.26	15.77±8.40	0.081
Phe	11.47±5.62	13.10±6.04	10.16±7.17	18.27±8.76	10.14±4.98	10.88±5.08	0.249
Leu	12.37±6.88	13.99±6.12	12.39±10.08	22.92±11.40	11.05±6.46	12.15±6.22	0.153
Trp	3.95±1.85	5.16±2.36	4.33±3.62	6.73±3.26	4.29±2.43	4.17±2.26	0.495
Thr	16.47±9.89	29.08±16.00	15.44±11.70	24.20±13.47	16.34±7.54	14.99±6.11	0.191
NEAA							
Ala	59.06±15.68	59.98±14.60	57.08±26.61	67.74±15.33	49.64±13.19	49.80±9.62	0.429
Arg	5.57±2.70	6.60±3.09	5.45±3.98	9.55±3.47	5.20±2.31	5.61±1.68	0.134
Asn	6.43±3.80	6.60±3.40	5.85±4.70	10.14±5.89	5.00±2.78	5.11±1.77	0.261
Asp	11.33±5.40	13.17±4.57	12.57±10.78	18.06±9.32	10.98±7.56	11.05±7.48	0.617
Cit	$0.24±0.14^{b}$	$0.24±0.07^{b}$	$0.28±0.15^{b}$	$0.41±0.13^{a}$	$0.20±0.04^{b}$	$0.26±0.09^{b}$	0.033
Cys	9.44±4.72	12.21±2.56	10.80±4.03	13.00±2.40	10.90±2.26	9.21±4.33	0.394
Glu	63.09±43.51	60.44±24.98	53.60±35.01	77.21±37.87	48.23±25.31	54.02±21.68	0.704
Gln	10.47±5.95	10.59±3.21	9.22±6.26	11.81±5.66	8.04±3.81	9.05±2.98	0.798
Gly	4.02±2.70	3.61±1.78	3.31±2.68	5.73±2.49	2.98±1.44	3.46±1.81	0.339
Pro	9.19±5.54	9.89±5.58	6.48±5.55	9.19±3.73	7.76±6.42	6.72±2.98	0.796
Ser	15.98±6.51	16.48±6.74	12.27±7.89	16.09±7.38	10.57±4.06	12.14±1.49	0.444
Tyr	15.83±6.99	16.94±8.01	14.50±12.11	26.28±12.91	13.56±5.98	13.51±6.03	0.166
The	0.003±0.002	0.001±0.001	0.004±0.004	0.005±0.005	0.001±0.001	0.004±0.002	0.752
GABA	0.34±0.10	0.40±0.13	0.56±0.37	0.44±0.19	0.30±0.07	0.32±0.18	0.241
Orn	1.58±0.27	1.69±0.60	1.51±0.45	2.03±0.21	1.49±0.30	1.44±0.19	0.090
His	6.01±1.64	6.20±1.54	5.54±2.73	6.76±1.75	5.09±1.09	5.76±1.46	0.677

Table S3 Effect of dietary MSCP on the content of fatty acids in breast muscle of Wenchang chickens ug/g.

Tratis	Groups						*P*-value
	Con	CTC	$PMSC_{400}$	$PMSC_{800}$	$PMSC_{1600}$	$PMSC_{3200}$	
c12:0	7.12±0.61^{c}	9.26±1.43^{a}	8.65±0.58^{ab}	8.08±1.57^{abc}	7.62±1.08^{abc}	8.31±1.16^{abc}	0.041
c16:0	1764.92±180.36	2149.42±209.33	2048.92±217.20	1941.92±560.10	1918.92±336.63	2060.58±210.88	0.378
c15:0	10.74±0.74	12.54±1.37	11.51±0.74	11.58±1.80	10.67±1.55	11.58±1.30	0.181
c18:0	594.83±49.98	639.33±69.04	661.25±44.88	631.17±111.12	583.92±77.84	662.33±74.24	0.340
c20:0	10.17±0.60^{bc}	10.64±0.34^{b}	10.67±0.45^{b}	11.62±1.44^{a}	9.48±0.70^{c}	10.56±0.67^{b}	0.002
c24:0	4.02±1.81^{b}	8.02±0.99^{a}	7.68±0.87^{a}	9.02±0.65^{a}	6.49±0.52^{a}	7.66±0.52^{a}	0.003
c14:0	57.67±3.82	72.50±7.92	69.50±9.19	66.00±15.20	64.33±10.87	67.92±5.92	0.162
c22:0	8.55±4.24^{ab}	10.58±1.10^{a}	5.28±2.38^{bc}	1.73±1.72^{c}	9.36±0.78^{ab}	8.89±4.47^{ab}	0.005
c17:0	13.30±1.30^{bc}	14.99±0.99^{ab}	14.68±0.91^{abc}	15.99±3.17^{a}	12.72±1.36^{c}	14.58±1.13^{abc}	0.025
c13:0	4.43±0.04^{a}	4.42±0.11^{a}	4.31±0.24^{ab}	4.17±0.26^{b}	4.08±0.19^{b}	4.16±0.25^{b}	0.019
c8:0	2.00±1.44	2.92±1.10	1.93±0.53	1.85±1.12	2.32±1.07	2.08±0.42	0.484
c10:0	3.64±0.16	3.97±0.25	3.88±0.20	3.82±0.38	3.75±0.27	3.87±0.21	0.336
c20:1	15.22±1.96	16.28±2.75	16.02±1.45	15.62±3.19	15.83±2.50	17.48±2.06	0.675
c16:1	191.42±37.50	230.92±42.92	226.58±70.38	194.67±45.12	219.33±56.61	233.51±58.34	0.806
c18:3n6	12.32±0.90	15.69±1.41	13.67±1.77	14.33±3.14	13.33±2.33	14.64±1.54	0.097
c20:5n3c	64.08±7.64	60.33±5.41	64.08±6.46	65.17±6.47	58.50±6.26	62.92±9.73	0.573
c18:2n6t	132.50±18.98	161.58±24.59	158.50±18.38	145.92±38.93	149.58±27.75	156.00±14.71	0.390
c20:3n6	86.08±5.36	86.42±9.04	87.58±8.17	93.58±11.03	82.58±6.57	89.67±13.74	0.473
c22:6n3c	116.83±21.68	112.58±24.19	121.08±29.97	98.33±13.45	97.25±11.57	112.42±15.25	0.259
c20:4n6	527.50±55.64	529.33±72.07	610.92±55.79	563.42±133.82	499.50±91.19	620.42±66.18	0.101
c18:3n3	769.33±128.92	978.58±190.73	912.67±128.31	809.58±259.94	848.50±176.03	890.00±99.61	0.360
c18:2n6	853.08±87.97^{b}	1009.17±52.05^{ab}	1009.33±52.05^{ab}	1032.83±246.12^{a}	863.75±97.71^{b}	993.33±70.80^{ab}	0.048
c18:1n9t	202.25±24.36	229.92±49.47	242.42±56.42	176.75±45.54	212.83±42.92	222.25±20.52	0.141
c22:1n9	/	/	/	4.86±3.09	8.28±1.70	/	0.001
c18:1n9c	304.92±52.89	388.25±73.28	364.33±48.70	328.83±100.80	330.08±63.44	351.67±36.39	0.328
